# Are There Multiple Visual Short-Term Memory Stores?

**DOI:** 10.1371/journal.pone.0001699

**Published:** 2008-02-27

**Authors:** Ilja G. Sligte, H. Steven Scholte, Victor A. F. Lamme

**Affiliations:** 1 Cognitive Neuroscience Group, Department of Psychology, University of Amsterdam, Amsterdam, The Netherlands; 2 Netherlands Institute for Neuroscience, Royal Netherlands Academy of Arts and Sciences (KNAW), Amsterdam, The Netherlands; University of Minnesota, United States of America

## Abstract

**Background:**

Classic work on visual short-term memory (VSTM) suggests that people store a limited amount of items for subsequent report. However, when human observers are cued to shift attention to one item in VSTM during retention, it seems as if there is a much larger representation, which keeps additional items in a more fragile VSTM store. Thus far, it is not clear whether the capacity of this fragile VSTM store indeed exceeds the traditional capacity limits of VSTM. The current experiments address this issue and explore the capacity, stability, and duration of fragile VSTM representations.

**Methodology/Principal Findings:**

We presented cues in a *change-detection task* either just after off-set of the memory array (*iconic-cue*), 1,000 ms after off-set of the memory array (*retro-cue*) or after on-set of the probe array (*post-cue*). We observed three stages in visual information processing 1) iconic memory with unlimited capacity, 2) a four seconds lasting fragile VSTM store with a capacity that is at least a factor of two higher than 3) the robust and capacity-limited form of VSTM. Iconic memory seemed to depend on the strength of the positive after-image resulting from the memory display and was virtually absent under conditions of isoluminance or when intervening light masks were presented. This suggests that iconic memory is driven by prolonged retinal activation beyond stimulus duration. Fragile VSTM representations were not affected by light masks, but were completely overwritten by irrelevant pattern masks that spatially overlapped the memory array.

**Conclusions/Significance:**

We find that immediately after a stimulus has disappeared from view, subjects can still access information from iconic memory because they can see an after-image of the display. After that period, human observers can still access a substantial, but somewhat more limited amount of information from a high-capacity, but fragile VSTM that is overwritten when new items are presented to the eyes. What is left after that is the traditional VSTM store, with a limit of about four objects. We conclude that human observers store more sustained representations than is evident from standard change detection tasks and that these representations can be accessed at will.

## Introduction

Humans are constantly interacting with a complex and ever-changing environment. Selectively orienting our attention to specific parts of the external world seems to be essential to efficiently process all available information. Although we tend to believe that we perceive everything around us, the visual *change-detection task* strikingly demonstrates that this is not the case. In the typical *change-detection task*, observers are shown a multi-item memory array (or a complex natural scene containing many items) and they are asked to remember as much individual items as possible. A short while after disappearance of the memory array, a probe array appears and subjects report whether the probe array is identical to the memory array or not. Observers are generally good at this task when they have to remember four items or less, but performance deteriorates rapidly when more than four items are shown in the memory array. A well-accepted explanation for this result is that people can store a maximum of about four integrated objects in visual short-term memory (VSTM) [1]–[7], although the exact capacity seems to depend on stimulus complexity [8]–[10] and the organization of objects in the memory array [11], [12].

Recently, several authors have begun to question whether indeed mental representation are limited to the four objects stored in VSTM. They probed for additional representations by introducing cues during the retention interval of a *change-detection task* that retrospectively indicate which item has to be attended (a so-called *retro-cue*). This is very similar to the way iconic memory is measured [13], only now the *retro-cue* is provided well beyond the time domain in which iconic memory can exert its influence. All experiments so far [14]–[20] have reported an increase in performance when a *retro-cue* is provided compared to when no cue or a cue during the probe array is provided (a so-called *post-cue*). This suggests that VSTM has an additional capacity that is however overwritten as soon as a second array (i.e. the probe array) is shown.

One can ask what happens to a VSTM representation when it is cued retrospectively. It seems that a *retro-cue* protects a fragile VSTM representation from interference with new information (such as the probe array), regardless of whether this new information is irrelevant [18] or task-relevant, [19], [20]. It does so by recruiting the same fron toparietal network (responsible for selective attention) as when a cue is shown before the presentation of an image [14], [16], [17], resulting in enhanced activity of the representation in object-specific cortex [17]. Most likely, this enhancement in object-specific cortex protects relatively fragile VSTM representations against overwriting. So, contrary to dogmatic views of VSTM as a robust and capacity-limited store that is able to retain information as long as subjects concentrate on the task at hand, VSTM seems to exhibit gradations of robustness, depending on the amount of attention that is allocated to it.

However, whether the capacity of VSTM indeed surpasses the “magical number 4” is still controversial [21] . All the findings referred to above are limited since the number of items presented in the memory array cannot be presumed to have really exceeded the capacity limits of VSTM, except maybe for the experiment of Landman [15]. Yet, a commonly heard objection against the high-capacity results of Landman is that oriented rectangles were used in the paradigm and subjects could have grouped these objects to form fewer compound figures (‘chunking’), hence the high capacity measure. Therefore, the current experiments further explore the capacity, stability and lifetime of fragile VSTM representations. In addition, we address whether ‘chunking’ of simple oriented rectangles into fewer compound figures can explain the high capacity of fragile VSTM.

## Results

### Representational limits in VSTM

**Figure 1:**
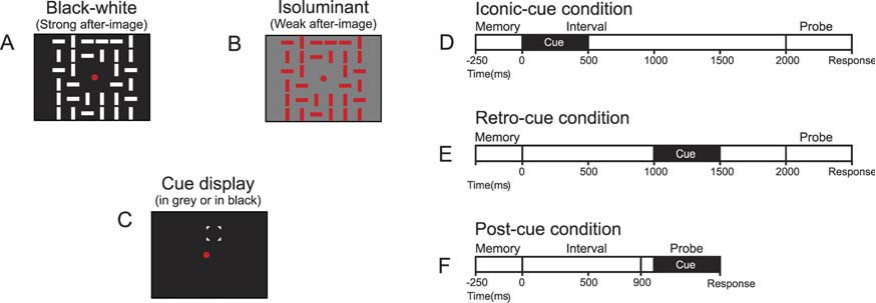
Design *Representational limits in VSTM.* A. High contrast black-white stimulus producing strong after-image B. Isoluminant red-grey stimulus producing weak after-image C. Cue display; background is black in high-contrast condition and grey in isoluminant condition D. Iconic-cue condition measuring iconic memory E. Retro-cue condition measuring fragile VSTM F. Post-cue condition measuring robust VSTM

The goal of this experiment was to produce estimates of the representational limits in VSTM and we varied set size of the memory array up to 32 figures accordingly. We used simple oriented rectangles, as the capacity of VSTM tends to decrease with stimulus complexity (see below). In the basic design, subjects were asked to detect changes between a briefly presented memory array (**Fig. 1a/b**) and a subsequently delivered probe array. There was a change in 50% of cases, and we cued the location of the potentially changing item at different moments after presentation of the memory array. This cue (**Fig. 1c**) was provided either 10 ms after off-set of the memory array (*iconic-cue* **;** **Fig. 1d**), 1,000 ms after off-set memory array, but before on-set of the probe array (*retro-cue* **;** **Fig. 1e**) or 100 ms after on-set of the probe array (*post-cue* **;** **Fig. 1f**). To prevent that subjects used a strategy of grouping similar items together, we rotated all other items by 90 degrees between memory and probe array. When subjects did use strategy of grouping, this should lead to the general percept of change on each trial, and a corresponding decrease in performance. In addition, we manipulated strength of the positive after-image by using either white items on a black background, or red items on an isoluminant gray background (**Fig. 1a/b**). By using white rectangles on a black background, we recruited both rod and cone systems, and by using red rectangles on a grey background of the same luminance, we recruited the isolated cone system [22].

It is well known that rod receptors integrate information over relatively long periods of time and will continue to respond for some period of time after off-set of a stimulus, whereas cone receptors respond to stimulation with very brief bursts of activation. In effect, by selectively recruiting the rod system, we induced a strong (**Fig. 1a**) or a weak (**Fig. 1b**), positive after-image. Still, the visibility of a strong, positive after-image would not last more than a few hundred milliseconds, and would thus only influence results when an *iconic-cue* is delivered (we measured phosphor persistence of our monitor to preclude that experimental effects were due to persistence of the display instead of persistence in the visual system, see **Materials and Methods**).

**Figure 2:**
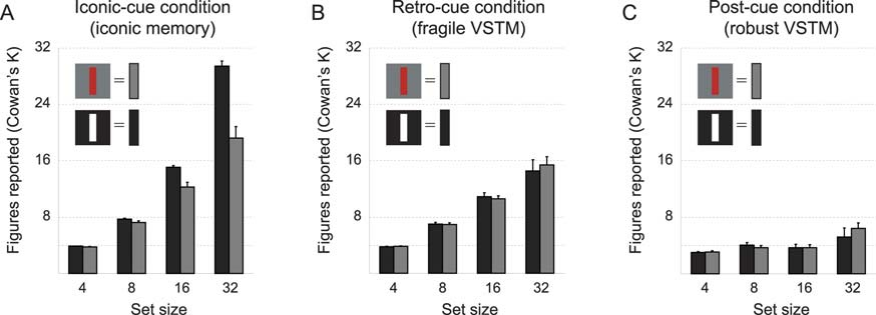
Results *Representational limits in VSTM.* A. Capacity of iconic memory depends on strength of after-image; unlimited for strong after-images and lower, but still high for weak after-images. B. Capacity of fragile VSTM not dependent on strength of after-image; capacity high for both kinds of stimuli. C. Capacity of robust VSTM not dependent on strength of after-image; capacity more or less limited to about 4 figures. Data are plotted as mean Cowan's K+SEM.

When *iconic-cues* (**Fig. 1d**) were delivered, subjects performed nearly perfectly regardless of set size when stimuli producing strong after-images were presented. However, performance was significantly worse when stimuli producing weak after-images were presented [F(1,9) = 22.36, p = .001] (**Fig. 2a**). On the other hand, when *retro-cues* (**Fig. 1e**) or *post-cues* (**Fig. 1f**) were delivered, no differences due to the strength of after-images were found [F(1,9) = .25, p = .63]. Still, we observed that subjects could report much more items when *retro-cues* were provided (**Fig. 2b**) instead of *post-cues* (**Fig. 2c**) [F(3, 7) = 38.45, p<.001].

Apparently, subjects can retain and report large amounts of information up to 1,000 ms after stimulus off-set, and this is not due to a retinal afterimage producing iconic memory. However, upon arrival of the next image we see ‘overwriting’ of this large capacity store and subjects can only access objects that are represented in VSTM in a robust way. Surprisingly, we found that positive after-images made up the majority of the iconic memory effect, but after-images do not seem to boost performance 1,000 ms after image off-set or after onset of a new image. Based on these results, we can say that there is evidence for two high-capacity stages in visual information processing: 1) iconic memory that is highly dependent on after-images of the shown image and does not seem to be limited in capacity (at least up to 32 objects), and 2) a fragile form of VSTM that at least exceeds a capacity of 10 objects on top of robust VSTM.

### Stability of VSTM representations

**Figure 3:**
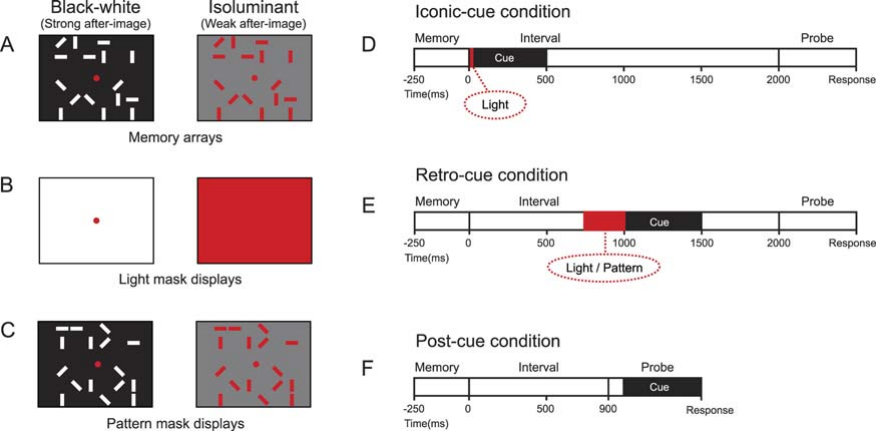
Design *Stability of VSTM representations.* A. Memory arrays. B. Light masks. C. Pattern masks; objects are at the same location as memory array, orientations are randomized. D. Iconic-cue condition with or without preceding 10-ms light mask. E. Retro-cue condition with or without preceding 250-ms light or 250-ms pattern mask. F. Post-cue condition.

We explored the stability of iconic memory and fragile VSTM representations. This was done by displaying masks before the attention-directing cue was presented. Subjects were informed of these irrelevant mask displays, and they were instructed to ignore them. The mask display was either: 1) a uniform display of light (**Fig. 3b**) in the same color as the previously shown objects in the memory arrray (**Fig. 3a**), or 2) a pattern mask that was identical to the previously shown memory array with respect to the location of all items, only orientation of individual items was randomly re-assigned (**Fig. 3c**). Again, in this experiment, all non-cued items were rotated between memory and probe array to prevent grouping.

**Figure 4:**
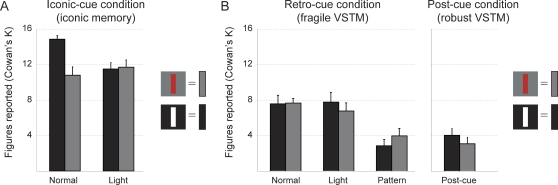
Results *Stability of VSTM representations.* A. Iconic memory is overwritten by presenting a light mask. B. Fragile VSTM is not influenced by the presence of a light mask, but a pattern mask erases fragile VSTM representations leading to drop-off in performance to robust VSTM levels. Data are plotted as mean Cowan's K+SEM.

The presentation of a light mask before the *iconic-cue* wiped out differences in capacity due to the strength of the after-image [F(1,9) = 18,99, p = .002] (**Fig. 4a**). Conversely, this manipulation did not affect performance in the *retro-cue* condition [F(1,9) = .17, p = .69] (**Fig. 4b**). Yet, the appearance of a pattern mask before the *retro-cue* did result in a dramatic performance deterioration [F(1,9) = 24.39, p = .001] such that no performance difference with the *post-cue* condition was observed [F(1,9) = .00, p = 1.00] (**Fig. 4b**).

The fact that iconic memory can be wiped out by a non-informational flash of light suggests that this type of memory is pre-categorical in nature and must be driven by persistent activation in the retina. Conversely, the same light mask did not influence *retro-cue* aided VSTM performance. Only when new, but irrelevant oriented rectangles were presented, did we observe that *retro-cues* could no longer aid standard VSTM performance. We suggest that iconic memory is driven by persistent retinal activation beyond stimulus duration, while persistent activation in visual and temporal cortex (without additional input of the retina) is responsible for maintenance of fragile VSTM representations (see also [17]).

### Influence of perceptual organization on change detection

A potential problem with our results thus far, showing higher capacity representations upon the *retro-cue* compared to the *post-cue,* is that our measure to counter chunking (rotating all irrelevant items between memory and probe arrays) could have introduced a difference in capacity by itself. It was recently shown that the orientation of lines is automatically coded in a memory representation [11], [12]. When the context of the item to report is changed (as in our case), retrieval is impaired. Only when attention is directed to the relevant item before the change occurs, it was observed that context changes did not affect performance. This alternative explanation could account for the higher performance in our *iconic-cue* and *retro-cue* conditions and a reduced performance in our *post-cue* condition (although, if VSTM is indeed limited to four items, this difference should have revealed it self as a lower-than-four capacity in the *post-cue* condition, not so much as a higher-than-four capacity in the *retro-cue* condition).

**Figure 5:**
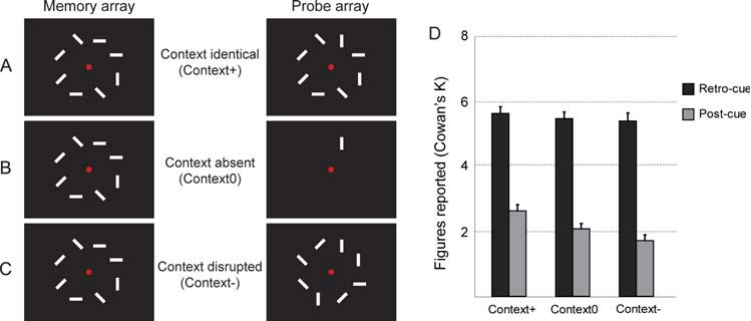
*Influence of perceptual organization on change detection.* A. Identical perceptual organization between memory and probe array. B. Perceptual organization is absent in probe array. C. Perceptual organization is disrupted between memory and probe array. D. A change in perceptual organization between memory and probe array does not influence the capacity of fragile VSTM, but it slightly reduces capacity of robust VSTM. Data are plotted as mean Cowan's K+SEM.

Here, we manipulated perceptual organization between memory and probe array to control for this alternative explanation; perceptual organization was either identical between memory and probe array (context+; **Fig. 5a**), absent since only the cued item was shown (context0; **Fig. 5b**) or disrupted since all non-cued items were changed between memory and probe array (context−; **Fig. 5c**). We only measured the *retro-cue* and *post-cue* conditions in this experiment.

A change in perceptual organization clearly influenced task performance in the *post-cue* condition (F(2,38) = 12.75, p<.001), but not in the *retro-cue* condition (F(2, 38) = 1.181, p = .177) (**Fig. 5d**). When we compared performance in the *retro-cue* condition with performance in the *post-cue* condition, the effect size increased as contextual information decreased (**context+:** *d* = 2.31; **context0:** *d* = 2.88; **context−:** *d* = 2.77). Thus, the difference in performance between the *post-cue* and the *retro-cue* conditions in the previous experiments was inflated by about 20 percent when the perceptual organization of the probe array is disrupted.

A change in the perceptual organization of the probe array reduces performance on a change detection task when attention is divided among multiple items as in our post-cue condition, but not when attention is focused on a single item (even when focusing of attention occurs retrospectively). Altogether, we conclude that differential use of context slightly inflates the capacity difference between the retro-cue and post-cue conditions, but the majority of the difference cannot be explained by this factor.

### Capacity of VSTM for complex objects

**Figure 6:**
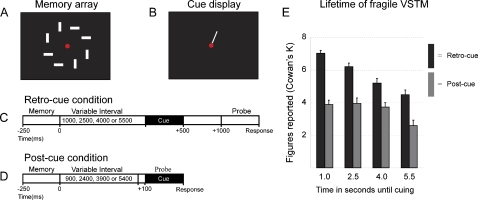
*Capacity of VSTM for complex objects.* A. Memory array with alphanumeric stimuli. B. Memory array with horoscope stimuli. C. Cue display. D. Retro-cue condition measuring fragile VSTM E. Post-cue condition measuring robust VSTM. F. Capacity of fragile VSTM is about twice the capacity of robust VSTM regardless of stimulus complexity. Rectangle data are adopted from Exp. 1 with set size 8. Data are plotted as mean Cowan's K+SEM.

In the previous control experiment, we assessed that differential grouping effects could explain about 20 percent of the difference in performance between *retro-cue* and *post-cue* conditions in Experiments 1 and 2. In the present experiment, more complex stimuli (either eight alphanumeric or eight horoscope characters; **Fig. 6a/b**) were employed in a similar design to the previous experiments. These kinds of stimuli cannot be (easily) ‘chunked’. If performance in the *retro-cue* condition is about twice the performance in the *post-cue* condition for complex stimuli (as it was for simple stimuli in the previous experiments), this would yield additional evidence that the effects of ‘chunking’ in the previous experiments are minor.

We found superior performance for the *retro-cue* condition (**Fig. 6d**) compared to the *post-cue* condition (**Fig. 6e**) for both alphanumeric [t(9) = 7.09, p<.001] and horoscope characters [t(9) = 5.38, p<.001] (**Fig. 6f**). For clarity, we have plotted the results of Experiment 1 with set size 8 in the same figure.

The capacity of fragile VSTM is always about twice the capacity of robust VSTM regardless of object type and complexity (as it was in Experiment 1 at set size 8). Capacity of both fragile and robust VSTM decreases as object complexity increases, which can be expected from previous experiments [8]–[10]. We conclude, based on this experiment and the previous control experiment, that the high estimates for fragile VSTM capacity in Experiment 1 and 2 cannot be explained by differential ‘chunking’ mechanisms between conditions.

### Lifetime of VSTM representations

**Figure 7:**
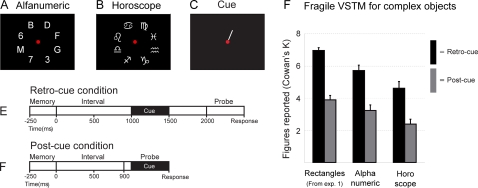
Lifetime of VSTM representations. A. Memory array. B. Cue display. C. Retro-cue condition measuring fragile VSTM with variable blank interval of 1000, 2500, 4000 or 5500 ms until cueing. D. Post-cue condition measuring robust VSTM with variable blank interval of 900, 2400, 3900 or 5400 ms until cueing. E. High-capacity, fragile VSTM decays linearly over time, whereas limited-capacity, robust VSTM is more or less durable. Data are plotted as mean Cowan's K+SEM.

We (previous sections) and others [14]–[20] observed that performance in the *retro-cue* condition did not drop to the performance observed in the *post-cue* condition even after 1,000 ms after display off-set. Here, we increased cue latencies up to 5.5 s after display off-set (**fig. 7c/d**) to find when *retro-cue* performance drops to the level of *post-cue* performance.

The high-capacity *retro-cue* performance decayed over time [F(1,19) = 102.61, p<.001], and the limited-capacity *post-cue* performance was stable until four seconds after stimulus off-set [F(1,19) = .23, p = .64] (**Fig. 7e**). Contrary to our expectations, we observed a drop in *post-cue* performance at the longest cue latency [F(3,17) = 8.524, p <.01]. Performance was significantly higher at all cue latencies when a *retro-cue* was shown compared to when a *post-cue* was shown (smallest t-value [t(19) = 7.92, p<.001]).

Fragile VSTM representations seem to exist for four seconds after stimulus off-set, at least. We are not sure if we can uniformly interpret the superior performance at the longest cue latency as evidence for the existence of fragile VSTM representations. At this cue latency, we see a drop-off in capacity for robust VSTM, possibly due to problems maintaining concentration. If subjects could maintain concentration for these long intervals, equal capacities for both stores might have been found. Thus, we conclude that fragile VSTM representations exist for a minimum of four seconds after stimulus off-set on top of robust VSTM.

## Discussion

Traditional work on visual short-term memory (VSTM) suggests that we can be aware of four visual objects only [1]–[7]. Does this suggest that we build up a limited internal picture of the world? Or can it be that visual scenes are more fully represented on a neural level, but not completely transferred to a reportable stage [23]–[25]? To answer this question, we used a *change detection task* in which attention-directing cues are incorporated. These cues retrospectively indicate which item has to be attended. We found that human observers can represent and access more objects than they can keep in traditional visual short-term memory (VSTM) up to four seconds after disappearance of the visual scene. Moreover, this high representational capacity is not due to iconic memory and seems to depend on the complexity of the observed objects.

### Three stages in visual information processing

By manipulating after-images and masks, we observed three stages in visual information processing; 1) iconic memory with unlimited capacity, 2) a long-lasting, but fragile form of VSTM with a capacity that is at least a factor 2 higher than the 3) robust form of VSTM that is clearly capacity-limited. Surprisingly, iconic memory representations seemed to depend on positive after-images of the previously shown image. When after-images were weak or when after-images were overwritten by flashes of light, iconic memory was found to be almost non-existent suggesting that it is primarily driven by persistent retinal activation beyond stimulus duration. The fragile form of VSTM was unaffected by the delivery of a light mask, but was completely overwritten to the level of robust VSTM by an irrelevant mask containing similar objects as the memory array. The capacity of both the fragile and the robust form of VSTM seemed to depend on stimulus complexity, which can be expected when we compare these results to previous findings [8]–[10].

### Chunking effects do not explain high-capacity measures of fragile VSTM

As far as we know, this paper and the paper of Landman [15] are the first to show the existence of a high-capacity, but fragile VSTM store on top of robust VSTM. A commonly heard objection against the high-capacity results of Landman (and thus against our results) is that oriented rectangles were used in the paradigm and subjects could have grouped these objects to form fewer compound figures (‘chunking’), resulting in an apparent high capacity. Indeed, it was recently shown that grouping of these kinds of stimuli may occur automatically [11], and this principle could reduce our set sizes to some smaller number. However, we are firm that this cannot explain the high-capacity results.

First, chunking in itself would not account for the difference in capacity that is found between *retro-* and *post-cue* conditions. If subjects would chunk items, this would increase the capacity of both fragile and durable VSTM. We found a capacity of about four objects for durable VSTM (the post-cue condition), which is well in accordance with traditional estimates. Second, to counter chunking in the current experiments we rotated all irrelevant items between the memory and probe arrays. Employing a strategy of chunking in this case would be fully detrimental to performance in the post-cue condition, as a ‘change’ to the compound figure would always be detected, regardless of whether the cued item changed or not.

Of course it can be argued that our measure to counter chunking (rotating all irrelevant items between memory and probe arrays) could have introduced a difference in capacity between retro- and post-cue by itself (see results section of experiment 3); it has been shown that when the context of the item to report is changed (as in our case), retrieval is impaired [11]. Only when attention is focused on one item, context changes do not affect performance. This could account for a reduced performance in our *post-cue* condition compared to the *iconic-* and *retro-cue* conditions. We performed an additional experiment in which we manipulated perceptual organization between memory and probe array to test this alternative explanation. We found that differential use of context slightly inflates the capacity difference between the retro-cue and post-cue conditions, but the majority of the difference cannot be explained by this factor.

Finally, in Experiment 4 we employed complex stimuli that cannot be (easily) chunked. The capacity of fragile VSTM still was about twice the capacity of robust VSTM (as it was for oriented rectangles in Experiment 1). Altogether, it seems unlikely that the high-capacity findings found here and in the paper of Landman are due to grouping mechanisms.

### Can we equate fragile VSTM to a form of iconic memory?

We make a tri-partite division between iconic memory, fragile VSTM, and durable VSTM. However, these results can also be explained by pleading for a dissociation of iconic memory in a *retinal* and a *cortical* icon (and traditional, capacity limited VSTM). This interpretation resembles earlier theoretical claims of Coltheart [26] that iconic memory might consist of both 1) a visible persistent component (alike our finding that iconic memory resembles a positive after-image) and, 2) an informational persistent component (akin to our finding of additional information in the *retro-cue* condition compared to the *post-cue* condition). There are two arguments that prevent us from drawing this conclusion. First, our *retro-cues* were presented well beyond the time period in which iconic memory effects are traditionally found. In addition, a recent study [19] found that items in fragile VSTM are not stored in a retinotopic way, but in a spatiotopic way. On the premise that iconic memory is a retinotopic phenomenon, it seems hard to reconcile this property with iconic memory, but not with VSTM. Still, these arguments can be quelled based on the available literature.

In traditional iconic memory paradigms, items are only shown once and here items are shown twice: once during encoding and once during report. It is well known that errors in iconic memory are location errors and not intrusion errors, suggesting that the location of items is lost over time and not the identity of the objects [27]–[29]. Our paradigm very much reduces spatial uncertainties (by showing items twice at the same location) and we can, therefore, presume that it could capture iconic effects for a longer period. Also, some evidence exists [30] that iconic memory might consist of a fast, retinotopic buffer followed by a relatively slow, spatiotopic buffer in which the spatial relations among visual information is represented. However, the effects found in that experiment were small, and other authors have not found these effects. Altogether, it remains speculative whether we can equate fragile VSTM to a form of VSTM. Yet, this approach is interesting since it relates to a current controversy in conscious vision (see next section).

### A fleeting form of visual awareness without direct report

Neurophysiologic findings suggest that we can discern two modes of visual processing; the feed forward sweep (FFS), and recurrent processing (RP) [31]. By selectively disrupting RP, but leaving FFS intact it is observed that visual awareness never arises. This was shown by backward masking [32], by applying transcranial magnetic stimulation (TMS) to V1 [33], [34], and by inactivating higher visual areas [35], [36]. Even when there are sudden lapses in awareness, it is observed that RP is absent, whereas FFS is intact [37].

While RP thus seems to be necessary for conscious perception, current controversy hinges on the question whether RP is sufficient for conscious perception [23]–[25], or that consciousness only occurs in the case of widespread RP, which includes areas necessary for cognitive access and control, such as the prefrontal cortex [38]. What happens when RP is limited to the visual and temporal cortex? Do we have conscious experience without report or no experience at all?

Experiments on iconic memory and fragile VSTM are interesting exactly because of this controversy. Just as robust VSTM forms a window on reportable and directly accessible conscious percepts, iconic memory and fragile VSTM could form a window on ‘perception without immediate cognitive access’. Only when attention is re-directed to the right location, representations can presumably ‘jump’ over the report threshold.

There are two key issues which need to be addressed. The first would be to establish the perceptual rather than unconscious nature of these kinds of representations, and the evidence for this is growing. Previous experiments showed that objects in fragile VSTM are processed up to the level of figure-ground organization [39], and that features are perceptually bound into coherent object representations [15]. The second issue is to establish a link between fragile VSTM (and iconic memory) and recurrent cortical processing. Our current results provide some evidence for this latter issue, by showing evidence for a long-lasting, i.e. reverberating nature. Still, neurophysiologic measures will have to confirm this link.

## Materials and Methods

### Participants

Ten right-handed young adults (5 females) participated in Experiment 1, 2 and 4; 40 right-handed young adults (25 females) in Experiment 3; and 20 right-handed young adults (16 females) in Experiment 5. All subjects had normal or corrected-to-normal vision and no colour deficiencies and they participated as part of a study course or for financial compensation. All subjects gave their written informed consent to participate in either one experiment. All experiments were approved by the local ethics committee of the department of Psychology of the University of Amsterdam.

### Equipment

**Figure 8:**
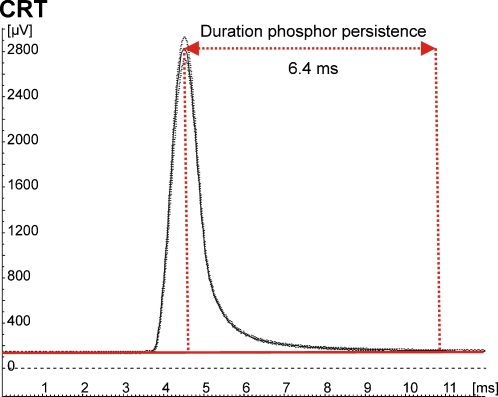
Phosphor persistence of CRT monitor. Phosphor persistence lasts approximately 6.4 ms after peak amplitude. Data are averages of 100 trials of single frame presentations of pure white light (87.66 cd/m^2^) presented at a refresh rate of 100 Hz. Each single frame presentation of light was followed by a 200-ms blank period.

All experiments were done on a 19 inch LG CRT-display (type FB915BP) at a refresh rate of 100 Hz. We measured phosphor persistence of the display using a photo-cell placed at the centre of the screen. Presented data are averages of 100 trials of single frames of pure white light (87.66 cd/m^2^) (see **Figure 8**). Each single-frame presentation was followed by a 200-ms blank period. It was observed that the phosphors returned to baseline activity approximately 6.4 ms after their peak amplitude. For all experiments, we used Presentation version 9.7 (NeuroBehavioral Systems, Inc.) to display our stimulation on the monitor.

### Experimental paradigm

#### Experiment 1

Stimulus displays consisted of grids of 36 locations that were each 2°×2° in size; total grid size 12°×12°. The centre four grid locations were always empty. Each display consisted of 4, 8, 16, or 32 rectangles with either a horizontal or a vertical orientation. Individual rectangles were 1.56°×0.39° in size and presented randomly at the centre of the grid locations–except for the 32 figures condition in which all locations were filled. All displays were composed of either pure white rectangles (87.66 cd/m^2^) on a pure black background (0.01 cd/m^2^; **Fig. 1a**)-or of red rectangles on an isoluminant gray background (both 13.52 cd/m^2^; **Fig. 1b**). Cues were composed of four white triangles–each 0.09°×0.09° in size-placed at the edges of a grid location (**Fig. 1c**). Subjects were seated 100 cm from a 19-inch display, which spanned 20.4 by 15.4 degrees of visual angle.

On each trial, we showed a 250-ms memory array containing 4 to 32 oriented rectangles. We instructed the subjects to remember as many oriented rectangles of the memory array as possible. On each trial, one rectangle was cued to indicate which item to report. After some delay, a probe array was shown and subjects were asked to indicate by button press whether the cued item had the same or different (50–50) orientation as the one shown in the memory array. Probe arrays were present until subjects made a response. All non-cued items were rotated by 90° to prevent subjects to use a strategy of encoding items in chunks. Cues were introduced at different latencies during the trial; either 10 ms after off-set of the memory array (*iconic-cue*; **Fig. 1d**), 1,000 ms after off-set of the memory array (*retro-cue*; **Fig. 1e**), or 100 ms after on-set of the probe array (*post-cue*; **Fig. 1f**). The interval between memory and probe array was 2000 ms for the *iconic-cue* and *retro-cue* conditions and 900 ms for the *post-cue* conditions. In effect, the *retro-cues* and *post-cues* were given at the same latency after memory array off-set ruling out differences in capacity due to a differential interval in which subjects had to remember all objects. Cue conditions of particular set sizes were presented in separate blocks of 64 trials each.

#### Experiment 2

Here, rectangles could have one of four possible orientations; horizontal, vertical, 45° to the vertical, and 135° to the vertical (**Fig. 3a**). Also, masks were introduced. Light masks were composed of uniform full-screen displays in red (13.52 cd/m^2^) or white (87.66 cd/m^2^) (**Fig. 3b**). Pattern masks were identical to the shown memory arrays with all elements at the same location, only orientation of individual rectangles was randomly re-assigned (**Fig. 3c**). We introduced a 10-ms light mask before the *iconic-cue* (**Fig. 3d**), a 250-ms light mask before the *retro-cue* or a 250-ms pattern mask before the *retro-cue* (**Fig. 3e**). Subjects were informed of the presence of mask displays and were instructed to ignore them. All other details were identical to Experiment 1.

#### Experiment 3

Displays consisted of eight rectangles–spanning 1.56°×0.39°-placed radially at 4 degrees of visual angle around the fixation point. The exact location of each rectangle was randomly jittered by half degree of visual angle towards the centre or the periphery. Rectangles could have one of four possible orientations; horizontal, vertical, 45° to the vertical, and 135° to the vertical. Only white rectangles (87.66 cd/m^2^) on a black background (0.01 cd/m^2^) were used. Cues consisted of a 3-pixel thick line which was at one end close (∼0.7°) to fixation and at the other end close (average ∼1.2°) to the critical item. We manipulated perceptual organization between memory and probe array; perceptual organization was either identical between memory and probe array (context+; **Fig. 5a**), absent since only the cued item was shown (context0; **Fig. 5b**) or disrupted since all non-cued items were changed between memory and probe array (context−; **Fig. 5c**). We only measured the *retro-cue* and *post-cue* conditions in this experiment. All other details were identical to Experiment 1.

#### Experiment 4

Displays consisted of either eight different alphanumerical symbols from a pool of 18 items (B,D,F,G,H,J,K,L,M,1,2,3,4,5,6,7,8,9) or eight different astrological symbols from a pool of 11 items (we excluded the symbol scorpio since it is very similar to the symbol virgo) placed radially at 4 degrees of visual angle around the fixation point (**Fig. 6a/b**). All symbols were presented at font size 64 in white (87.66 cd/m^2^) on a black background (0.01 cd/m^2^). Only the *retro-cue* condition (**Fig. 6d**) and the *post-cue* condition (**Fig. 6e**) were presented. All non-cued items were not changed between memory and probe array. All other details were identical to Experiment 3.

#### Experiment 5

Displays consisted of eight rectangles–spanning 1.56°×0.39°-placed radially at 4 degrees of visual angle around the fixation point (**Fig. 7a**). We presented only the *retro-cue* and *post-cue* conditions, and we varied the blank interval between memory and probe array between 1,000 and 5,500 ms for *retro-cue* conditions (**Fig. 7c**) and between 900 and 5,400 ms for *post-cue* conditions (**Fig. 7d**) in steps of 1,500 ms. All non-cued items were not changed between memory and probe array. All other details were identical to Experiment 3.

### Procedure

#### Experiment 1 & 2

Subjects were heavily trained on the task in a separate 3-hour session before entering the experimental sessions. In the practice session, all conditions at all set sizes were practiced at least once for the high-contrast stimuli, and subjects were allowed to practice blocks more than once when they indicated that they could have attained higher performance. Results obtained from subjects during the training sessions were qualitatively similar in the sense that iconic memory capacity was always much higher than working memory capacity. Training increased capacity for all conditions up to the ceiling levels reported in the results section. After the practice session, subjects participated in the experimental session with high-contrast stimuli first and subsequently in the session with isoluminant stimuli; this procedure was counterbalanced over subjects. Subjects were instructed to maintain fixation throughout the entire experiment, and they were encouraged to indicate changes only if they were certain that a change had occurred. The experiment was done in a darkened room to increase the strength of the after-images [22].

#### Experiment 3

Subjects were either assigned to the *retro-cue* condition first or to the *post-cue* condition first in a counterbalanced fashion. All different perceptual organizations (context+, context0, context−) were presented randomly intermixed within a block consisting of 48 trials. After doing a block of one condition (f.i. the retro-cue condition), subjects did a block of the other condition. This sequence was repeated five times, and the first block of each condition was discarded in the analysis since it functioned as a training block. Thus, subjects performed a total of 192 trials in each condition. All other details were identical to Experiment 1.

#### Experiment 4

Subjects were either assigned to the *retro-cue* condition first or to the *post-cue* condition first in a counterbalanced fashion. Alphanumerical versions were always performed first followed by the horoscope versions. This sequence was repeated three times, resulting in three sessions of 48 trials for each condition. The first block of each condition was discarded in the analysis since it functioned as a training block. All other details were identical to Experiment 1.

#### Experiment 5

Subjects practiced the *retro-cue* condition with an ISI of 1,000 ms and the *post-cue* condition with an ISI of 900 ms for 48 trials each. Subsequently, they entered the experimental condition in which they performed 48 trials on each condition. All conditions were randomly intermixed throughout the entire experiment. All other details were identical to Experiment 1.

### Data analysis

We computed memory capacity measures using a formula developed by Cowan [21]. The formula is *K* = (hit rate–0.5+correct rejection rate–0.5)*N, and gives an estimate of the representational capacity and corrects for guessing trials. All statistical analyses were performed with repeated measures ANOVAS. In some instances, we tested specific differences with paired t-tests.
